# Causes and Clinical Profiles of Ascites at University of Gondar Hospital, Northwest Ethiopia: Institution-Based Cross-Sectional Study

**DOI:** 10.1155/2019/5958032

**Published:** 2019-07-08

**Authors:** Oumer Abdu Muhie

**Affiliations:** Department of Internal Medicine, CMHS, University of Gondar, P.O. Box 196, Ethiopia

## Abstract

**Background:**

Ascites is a common clinical condition encountered by physicians in day-to-day practice. It is caused by various underlying diseases. Knowing the etiologies is vital because further investigations and definitive treatment largely rely on the specific disease entity considered.

**Objective:**

The aim of this study was to determine the epidemiology of causes of ascites and complications among patients with ascites from the medical department at the University of Gondar Hospital.

**Methods:**

Data on sociodemography, major symptoms, and signs, risk factors, past medical illnesses, and results of important investigations were collected using pretested questionnaires among all patients with ascites in the University of Gondar Hospital in a sample size of 52. Data were collected by well-trained physicians and analyzed by using SPSS 16. Results were depicted descriptively with measures of central tendency, dispersion, and using tables and graphs.

**Results:**

A total of 52 patients were included in this study from November 1, 2018 to March 30, 2019. Thirty (57.7%) of them were males and the majority (77%) of the participants were fifty years old or younger. The mean age was 43.8 (± 14). The majority (86.5%) of the participants were from a rural area. Thirty-eight (73%) patients take alcohol occasionally while 11(21.2%) patients take alcohol frequently or massively. Eight (15.4%) patients reported a history of multiple sexual partners. Herbal medicine use was reported by 28 patients (53.8%). Only 5 (9.6%) patients were overweight. Chronic liver disease (CLD) was the major cause of ascites in this study in 24 (46.2%) patients. The other main causes of ascites were heart failure from various causes (19.2%), tuberculosis and hepatosplenic schistosomiasis contributing to 11.5% each and chronic kidney disease (5.8%). Five (20.8%) CLD patients had spontaneous bacterial peritonitis as a complication. Five (20.8%) and 4 (16.7%) CLD patients had hepatocellular carcinoma and hepatic encephalopathy as complications, respectively. Nine (17.3%) patients had variceal bleeding; six of the patients were diagnosed to have CLD while the remaining patients were having hepatosplenic schistosomiasis.

**Conclusion:**

In conclusion, liver cirrhosis is the major cause of ascites in Gondar, Ethiopia, while chronic viral hepatitis infections (hepatitis B (HBV) and C (HCV) viruses) are the main causes of liver cirrhosis. The other major causes included heart failure, tuberculosis, and hepatosplenic schistosomiasis. It is wise to consider and give priority to these diseases whenever one is evaluating a patient with ascites.

## 1. Introduction

### 1.1. Statement of the Problem

Ascites, the collection of fluid in the peritoneal cavity, occurs with a variety of disease states. Abscess formation and localized or cystic accumulations are not included in the definition of this term [1]. Usually, it is a clinical finding and can be confirmed by a diagnostic paracentesis. Subclinical amount of fluid (i.e., less than 1.5 liters) can be detected using ultrasonography or computed tomography of the abdomen [2].

Ascites formation is the result of a series of anatomical, pathophysiological, and biochemical changes. The specific causes of ascites can be divided into those associated with portal hypertension (cirrhotic ascites) and those unrelated to portal hypertension (noncirrhotic ascites). In patients with liver cirrhosis, ascites develops as a consequence of sinusoidal portal hypertension, which results in alterations to capillary pressure, permeability and accumulation of retained fluid in the abdominal cavity. This mechanism of fluid accumulation is known as transudation. The passage of high molecular weight substances is limited because capillary damage is not the underlying process in transudation. Another mechanism of ascites formation is known as exudation; ascites development is secondary to increased vascular permeability due to the inflammatory process, tumoral invasion, or traumatic damage to the peritoneum or intraperitoneal organs [1].

Ascitic fluid may accumulate rapidly or gradually depending upon the cause. Mild ascites may not produce any symptoms. Moderate ascites may just produce an increase in abdominal girth and weight gain. Large amounts of fluid can produce abdominal discomfort and the appearance of hernias particularly umbilical hernia and hinder the mobility of the patient. Ascites can be the first sign of liver disease. Thus, it is important to obtain a history of risk factors for a liver disease like alcohol consumption, drug abuse, blood transfusions, or hepatitis in the past. The sudden development of ascites in a previously stable patient of cirrhosis should raise the suspicion of hepatoma [2].

Chronic hepatic schistosomiasis develops years later in young and middle-aged adults with a long duration of intense infection. Diffuse collage deposits in the periportal spaces lead to pathognomonic periportal or "Symmers pipestem fibrosis" with accompanying splenomegaly and portal hypertension. However, the hepatocellular function remains normal. Leading causes of morbidity and mortality include the formation of ascites and esophageal bleeding from varices [3].

Tuberculous peritonitis (TBP) is an uncommon presentation of TB without any other debilitating diseases such as cirrhosis, diabetes, and chronic renal failure on continuous ambulatory peritoneal dialysis [4].

Malignant ascites is defined as an accumulation of excess fluid in the peritoneal cavity secondary to a disseminated malignancy [5]. According to an estimate, it affects around 3.6-6% of patients admitted to palliative care [6]. Malignant ascites is an ominous sign with an average survival of around 20 weeks from its diagnosis [7].

The management of ascites largely depends on the cause. Hence identifying the cause ascites is of paramount importance. Diagnosis of the cause of ascites requires taking a thorough history, doing a meticulous physical examination and performing important investigations.

Laboratory evaluation should include liver biochemical testing, serum albumin level measurement and prothrombin time (PT) or international normalized ratio (INR) determination to assess hepatic function as well as a complete blood count (CBC) to evaluate for the presence of cytopenias that may result from portal hypertension or of leukocytosis, anemia, and thrombocytosis that may result from systemic infection. Serum amylase and lipase levels should be checked to evaluate the patient for acute pancreatitis. Urinary protein quantification is indicated when nephrotic syndrome, which may cause ascites, is suspected.

In selected cases, the hepatic venous pressure gradient (pressure across the liver between the portal and hepatic veins) can be measured via cannulation of the hepatic vein to confirm that ascites is caused by cirrhosis. In some cases, a liver biopsy may be necessary to confirm cirrhosis [8].

The etiology of the ascites is best determined by paracentesis, a bedside procedure in which a needle or small catheter is passed transcutaneously to extract ascitic fluid from the peritoneum. The ascitic fluid should be sent for measurement of albumin and total protein levels, white cell count with differentials, and, if an infection is suspected, Gram's stain and culture, with inoculation into blood culture bottles at the patient's bedside to maximize the yield. A serum albumin level should be measured simultaneously to permit the calculation of the serum-ascites albumin gradient (SAAG) [9].

Cytology can be useful in the diagnosis of peritoneal carcinomatosis. Tuberculous peritonitis is typically associated with ascitic fluid lymphocytosis but can be difficult to diagnose by paracentesis. A smear for acid-fast bacilli (AFB) has a diagnostic sensitivity of only 0 to 3%; a culture increases the sensitivity to 35–50%. In patients without cirrhosis, an elevated ascitic adenosine deaminase level has a sensitivity (SN) of >90% when a cut-off value of 30–45 U/L is used. When the cause of ascites remains uncertain, laparotomy or laparoscopy with peritoneal biopsies for histology and culture remains the gold standard [9].

An abdominal ultrasound is useful in confirming the presence of ascites and in the guidance of paracentesis. Both ultrasound and computerized tomography (CT) are useful in distinguishing between causes of the portal and nonportal hypertensive ascites. Doppler ultrasound and CT can detect thrombosis of the hepatic veins (Budd-Chiari syndrome) or portal veins. In patients with nonportal hypertensive ascites, these studies are useful in detecting lymphadenopathy and masses of the mesentery and of solid organs such as the liver, ovaries, and pancreas. Furthermore, they permit directed percutaneous needle biopsies of these lesions [8].

### 1.2. Literature Review

Various studies have shown different results regarding the causes of ascites in different parts of the world. Among these studies, some of them are mentioned in the following paragraphs.

Most patients (85%) with ascites in the United States have cirrhosis. The three most common causes of cirrhosis are excess alcohol use, chronic hepatitis C, and nonalcoholic steatohepatitis (NASH) related in many cases to obesity. As the obesity epidemic evolves, NASH could become the most common cause of cirrhosis. Many patients have 2 of these conditions, and some have all 3. In approximately 15% of patients with ascites, a nonhepatic cause of fluid retention is identified [10]. Ascites is the most common complication of cirrhosis, which is also the most common cause of ascites [1].

According to a study conducted in Italy among a series of 1155 consecutive patients (751 men; 404 women), 33% and 18% of the men were alcoholics and hepatitis B surface antigen (HBsAg) positive, respectively; and the corresponding figures for the women were 15% and 6%, respectively [11].

In a case-control study done to determine the causes of cryptogenic cirrhosis among 147 participants ( in 1:2 ratio), the prevalence of obesity (55% versus (vs.) 24%) and type II diabetes (47% vs. 22%) was significantly higher in patients with cryptogenic cirrhosis compared with controls. Twenty-three percent of patients with cryptogenic cirrhosis had both obesity and diabetes compared with 5% among controls (P =0.002) [12].

In a study performed to evaluate the role of ascitic albumin gradient versus the ascitic protein in differentiating portal and nonportal hypertensive causes of ascites among 901 patients, the albumin gradient correctly differentiated causes of ascites due to portal hypertension from those that were not due to portal hypertension in 96.7% of the time [13].

According to a study conducted in Pakistan among 50 patients with ascites, cirrhosis was the commonest cause contributing to 80% (40 patients) of the cases. The remaining causes were chronic renal failure 10%, peritoneal malignancy 4%, cardiac failure 2%, and peritoneal tuberculosis 4%. In this study 58% (29 patients) were female. Among the cirrhotic patients, hepatitis C infection contributed 80% while hepatitis B was seen in 12.5% of patients. Alcoholic liver disease was not reported as a cause. One patient had hepatocellular carcinoma [14].

In a trial done in Qatar among 104 patients with ascites with the majority (67%) of them being male, cirrhosis was the commonest cause of ascites contributing to 59.6 % of the cases. The other causes were malignant ascites in 12 patients (11.5%), malignancy related ascites in ten patients (9.6%), tuberculous peritonitis in eight patients (7.7%), heart failure in seven patients (6.7%), nephrotic syndrome in three patients (2.9%), chylous ascites in one patient (1.0%), and eosinophilic ascites in one patient (1.0%). Among the cirrhotic patients, chronic alcoholism was cause in 29 (46.7%) patients, while HBV and/or HCV were the causes in 24 (38.7%) patients. The remaining 9 (14.5%) patients had miscellaneous causes of cirrhosis [15].

In prospective study, conducted at Ibadan, Nigeria, among ninety adult patients with ascites, 40 (44%) had liver cirrhosis, 21 (23%) had tuberculous peritonitis, 20 (22%) had malignant ascites, 5 (6%) had heart diseases, and 4 (5%) had nephrotic syndrome [16].

In a study conducted in 1989 in Ethiopia among 240 patients for whom laparoscopic evaluation for patients with ascites and other abdominal conditions was done, 144 patients had ascites from which 82% were due to cirrhosis while 9% were due to tuberculosis peritonitis [17].

In a research conducted by Krastev et al. at 2013 among 167 patients with cirrhosis and ascites 25 patients had SBP and 22 patients had secondary bacterial peritonitis [18].

Mortality from ascites is approximately 15% in the first year and 44% by the fifth year, so referral for liver transplant evaluation is often indicated [19].

### 1.3. Significance of the Study

Ascites is a common clinical condition encountered by physicians in a day-to-day practice. It poses a diagnostic and therapeutic challenge to physicians because the causes are various. The causes are different in different settings. As to the best of our knowledge, there are no studies in Ethiopia addressing this particular issue. Knowing the epidemiology of the causes of ascites is very vital for physicians as it gives directions on what diseases to focus on for investigation and treatment.

## 2. Objectives

### 2.1. Primary Objective

To assess the causes of ascites, its complications, and factors associated with it among medical patients in University of Gondar Hospital.

### 2.2. Specific Objectives

To identify common causes of ascites.

To describe complications of ascites.

## 3. Methodology

### 3.1. Study Design

Institution-based cross-sectional study.

### 3.2. Study Area and Period

The study was conducted at the University of Gondar Hospital. The University of Gondar Hospital is one of the largest hospitals in the country. It is a tertiary teaching hospital serving a catchment area of approximately seven million peoples. It has different departments like internal medicine, surgery, pediatrics, gynecology/obstetrics, dermatology, ophthalmology, and so on. It has follow-up clinics for major chronic illnesses under the department of internal medicine. The majority of patients presenting with ascites will present to the medical outpatient department (OPD), medical emergency, and medical wards. The medical OPD, emergency, and wards are staffed with internists, internal medicine residents, interns, and nurses. The patient's clinical profile is kept recorded in their charts.

The study was conducted from November 1, 2018, to March 30, 2019.

### 3.3. Source Population

The source population was all adult patients with ascites who are visiting medical units at the University of Gondar Hospital.

### 3.4. Study Population

The study population was all adult patients with ascites who are visiting medical units at the University of Gondar Hospital during the study period.

### 3.5. Inclusion and Exclusion Criteria

#### 3.5.1. Inclusion Criteria

All adults aged greater than or equal to 18 years who have ascites.

#### 3.5.2. Exclusion Criteria

Critically ill patients and who are not able to communicate.

Those patients who failed to give consent to participate in the study.

### 3.6. Sample Size Determination

The sample size was not calculated; all patients who visited the University of Gondar Hospital with ascites and met the inclusion criteria are included.

#### 3.6.1. Sampling Procedure

The sampling procedure was a convenient sampling technique, where consecutive patients with ascites who present to medical units at the University of Gondar Hospital were included in the study.

### 3.7. Data Collection

Data were collected using pretested questionnaires which were composed of variables on sociodemographic characteristics, sexual history, weight and height, presence or absence of diabetes, chronic kidney disease, and presence or absence of known cardiac and liver disease. A family history of liver disease was also included. Drug history including herbal medicine and alcohol consumption was also included in the questionnaire. Data were collected by interviewing participants, reviewing records, and doing physical examinations. The data collectors and the investigator had no direct role in the care of patients included in this study.

### 3.8. Variables

- Ascites
- Causes of ascites
- Age
- Sex
- Residence
- Occupation
- Marital status
- Human-immunodeficiency virus (HIV) status
- Alcohol consumption
- Herbal medicine use
- Viral hepatitis (HBV; HCV)
- Diabetes mellitus
- chronic kidney disease
- family history of similar illness
- multiple sexual partners
- Body mass index (BMI)
- Blood transfusion

### 3.9. Operational Definitions

(i) Ascites:
  (a) Signs of fluid collection on abdominal physical examination
    (1) Positive Shifting dullness
    (2) Positive fluid thrill
  (b) Peritoneal fluid collection on abdominal ultrasonography
  (c) Previously documented ascites and patient taking diuretics
(ii) Cirrhotic ascites:
  (a) Ascites due to cirrhosis
(iii) Spontaneous bacterial peritonitis
  (a) Peritoneal fluid cell count: ≥ 500/ microL (> 50%, polymorphonuclear (PMLs)) or > 250/ microL PMLs and
  (b) Culture: positive for typical bacteria (Escherichia coli, Streptococcus pneumonia, and others)
(iv) Paracentesis: a procedure of taking ascitic fluid using a needle from the peritoneal cavity; preferably from the left lower quadrant.
(v) Alcohol consumption was classified as per patients' response to questions regarding the frequency of alcohol intake and the effects of alcohol as follows:
  (a) None: no history of alcohol intake
  (b) Occasional: alcohol intake is not more than two days per week
  (c) Frequent: alcohol intake in at least three days per week
  (d) Massive: alcohol intake in at least three days per week and history of alcohol intoxication, a need for eye-opener, or history of untoward social consequence of alcohol (like divorce, loss of job, quarrel, etc.)

### 3.10. Data Compilation and Analysis

Data were collected using a pretested questionnaire among patients with ascites in the University of Gondar Hospital medical OPDs, medical emergency, and wards. It was collected by well-trained physicians and was supervised by the investigator for clarity and completeness. Data on sociodemographic characteristics, clinical parameters like icterus, parotid swelling, and signs of ascites and investigations profiles like viral markers (HBV; HCV), bilirubin level, albumin level, and abdominal ultrasound were collected.

The data were checked for completeness. Data were entered, compiled, and analyzed using statistical package for the social sciences (SPSS) 16. Descriptive statistics were generated on patient demographics, clinical parameters, frequencies of factors, and complications of ascites.

### 3.11. Ethical Consideration

Ethical clearance was obtained from the Institutional Ethical Review board of, College of Medicine and Health Sciences, University of Gondar. Confidentiality of all the data to be collected was seriously respected. Oral informed consent was obtained from each participant.

## 4. Result

### 4.1. Sociodemographic Characteristics

A total of 52 patients with ascites were included in this study. Thirty (57.7%) of them were males and the majority (77%) of the participants were fifty years old or younger. The mean age was 43.8 (± 14); the age range of the patients was from 19 to 80. The majority (86.5%) of the participants were from a rural area and 41 (78.8%) were married. Three-fourths of the patients were farmers.  Table 1 shows the sociodemographic characteristics of the study participants.

### 4.2. Risk Factors among Participants

Thirty-eight (73%) patients take alcohol occasionally while 11 (21.2%) patients take alcohol frequently or massively. Eight (15.4%) patients reported a history of multiple sexual partners. Only 2 patients reported contact with jaundiced persons. Herbal medicine use was reported by 28(53.8%); they reported the use of smokable, drinkable, and dabbable medicines. One patient was diabetic while 4 patients had a history of blood transfusion receipt. The details of the risk factors are shown in Table 2.

### 4.3. Clinical Profiles of Participants

The mean body mass index of the patients was 20.1 (±3.1) kg/m2. Five (9.6%) patients were overweight while 14 (29.6%) patients were malnourished with BMI ranging from 13.8kg/m2 to 18.1kg/m2. The physical findings of the study participants are summarized in Table 3.

### 4.4. Investigation Profiles of Participants

Ascitic fluid was analyzed in 25(48.1%) patients; however it was not analyzed in the remaining 51.9% patients. The ascitic white cell count ranged from no cell to 6600 cells per microliter. The mean ascitic white cell count was 673.6 (±1306.9). In 50% of the patients with ascitic fluid analysis, the neutrophil white cells were dominant greater than fifty percent of ascitic leukocyte count. Gram stain and acid-fast bacilli staining were done in 24 (46.2%) patients. Only 2 patients had positive gram stain findings while none had a positive AFB stain. The investigation profiles of the study participants are depicted in Table 4.

Complete blood count was measured in 49 (94.2%) patients. The white blood cell count ranged from 1200 per microliter to 294 000 per microliter; the mean WBC count was 18 224 (±55845.2). The mean hematocrit of the patients was 34.5% (±10.7%), which ranged from 9.2% to 57.3%. The mean platelet count was 182 776(13 000-496 000).

Alanine transaminase (ALT) and aspartate transaminase (AST) were determined in 31 and 23 patients, respectively. The mean ALT level was 44.1 (± 51.8) IU/L and the mean AST level was 54.9 (±47.5) IU/L. Serum albumin was determined in 23 patients and the mean albumin level was 2.8(±0.78) g/dl.

### 4.5. Chronic Liver Disease, Causes, and Complications

**Figure 1:**
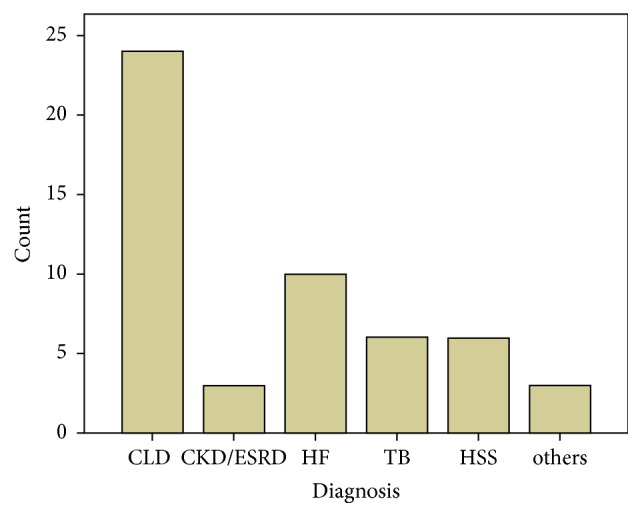
It shows the frequency of the different causes of ascites. CLD: chronic liver disease; CKD/ESRD: chronic kidney disease/end-stage renal disease; HF: heart failure; TB: tuberculosis; HSS: hepatosplenic schistosomiasis.

Chronic liver disease was the major cause of ascites in this study, 24 (46.2%). The other main causes of ascites were heart failure from various causes (19.2%), tuberculosis, and hepatosplenic schistosomiasis contributing to 11.5% each and chronic kidney disease (5.8%). Other causes were chronic myelogenous leukemia (in 2 patients) and visceral leishmaniasis (1 patient). The causes of ascites and their frequency are shown in bar Figure 1.

Chronic hepatitis B virus infection was the leading cause of CLD in this study contributing to 34.8% of the CLDs. The other causes of CLD were HCV infection (13%) and alcoholic liver disease (16.7%). The cause of CLD was Wilson's disease in one patient while none of the chronic liver diseases was due to nonalcoholic steatohepatitis.

Five (20.8%) CLD patients had spontaneous bacterial peritonitis as a complication. Five (20.8%) and 4 (16.7%) CLD patients had hepatocellular carcinoma and hepatic encephalopathy as complications, respectively. Generally, nine (17.3%) patients had variceal bleeding; six of the patients were diagnosed to have CLD while the remaining patients were having hepatosplenic schistosomiasis.

Moderate to severe thrombocytopenia as defined by platelet count of less than 100, 000 per microliter was more common in hepatosplenic schistosomiasis (4 out of 5 patients) and in cirrhotic patients (7 out of 23 patients). However, none of the other causes of ascites has caused moderate to severe thrombocytopenia.

## 5. Discussion

Ascites is a common clinical problem confronting a physician in Gondar, Ethiopia. Its incidence and prevalence are not enumerated in this set up before. Similarly, the prevalence of the causes of ascites has not been studied in Gondar, Ethiopia. Liver cirrhosis was found to be the most common cause of ascites in this study contributing to nearly half (46.2%) of the cases. Chronic viral hepatitis infections were the main causes of liver cirrhosis (47.8% of the liver cirrhosis). Alcoholic liver disease had contributed to 16.7% of the liver cirrhosis.

Liver cirrhosis was found to be the main cause of ascites in this study similar to other studies done elsewhere [10, 14–18]; however, the proportion of ascites caused by liver cirrhosis was lower as compared to the studies conducted in USA, Qatar, and Ethiopia [10, 15, 17]. This could be because of the higher tuberculosis and hepatosplenic schistosomiasis in the current study, where such infections are epidemic in the study setting. Nevertheless, the cirrhotic ascites rate was comparable to that of the studies done in Nigeria and Qatar [15, 16].

In contrast to studies done in Qatar [15] and USA [10] where alcoholic liver disease and NASH were more common causes of cirrhotic ascites, viral hepatitis especially HBV was the commonest cause of cirrhotic ascites in this study.

Heart failure, tuberculosis, and hepatosplenic schistosomiasis were relatively more common in this study in contrast to studies done in the USA, Qatar, and Pakistan [10, 14, 15]. However, tuberculosis incidence (11.5%) in this study was lower in contrast to the study done in Nigeria (22%) but was comparable to a previous Ethiopian study (9%).

Malignant related ascites was lower (in 2 patients) in this study in contrast to the studies done in Pakistan, Qatar, and Nigeria [14–16]. This is probably due to the younger age of the participants in this study where the majority of the participants are younger than 50 years of age.

Cirrhotic ascites patients had various complications like hepatocellular carcinoma (5 patients), spontaneous bacterial peritonitis (5 patients), and variceal hemorrhage (6 patients). Four cirrhotic patients had hepatic encephalopathy. Three of the six hepatosplenic schistosomiasis patients had variceal bleeding.

In conclusion, liver cirrhosis is the major cause of ascites in Gondar, Ethiopia, while chronic viral hepatitis infections (HBV and HCV) are the main causes of liver cirrhosis. The other major causes included heart failure, tuberculosis, and hepatosplenic schistosomiasis. It is wise to consider and give priority to these diseases whenever one is evaluating a patient with ascites.

## Figures and Tables

**Table 1:** Sociodemographic characteristics of study participants.

Factor		Frequency (%)
Age (years)	18-35	16(30.8)
	36-50	24(46.2)
	51-65	9(17.3)
	≥66	3(5.7)
Sex	Male	30(57.7)
	Female	22(42.3)
Residence	Urban	7(13.5)
	Rural	45(86.5)
Marital status	Married	41(78.8)
	Single	8(15.4)
	Divorced	1(1.9)
	Widowed	2(3.8)
Occupation	Farmer	39(75)
	Merchant	1(1.9)
	Government employee	2(3.8)
	Private employee	1(1.9)

**Table 2:** Risk factors for ascites among study participants.

Risk factor		Frequency (%)
History of alcohol ingestion	None	3(5.8)
	Occasional	38(73.1)
	Frequent	7(13.5)
	Massive (Alcohol dependent)	4(7.7)
History of multiple sexual partners	Yes	8(15.4)
	No	44(84.6)
Contact history to jaundiced patient	Yes	2(3.8)
	No	50(96.2)
History of herbal medicine use	None	24(46.2)
	Smokable	3(5.8)
	Drinkable	23(44.2)
	Dabbable	2(3.8)
Diabetes history	Yes	1(1.9)
	No	51(98.1)
History of blood transfusion	Yes	4(7.7)
	No	48(92.3)

**Table 3:** Physical findings of the patients.

Risk factor		Frequency (%)
Murmur	Yes	10(19.2)
	No	42(80.8)
Elevated JVP	Yes	14(26.9)
	No	38(73.1)
Pleural effusion	Yes	17(32.7)
	No	35(67.3)
Palpable/ballotable spleen	Yes	21(40.4)
	No	31(59.6)
Palpable/ballotable liver	Yes	13(25)
	No	39(75)
Peripheral edema	Yes	25(48.1)
	No	27(51.9)
Icterus	Yes	8(15.4)
	No	44(84.6)
Axillary hair loss	Yes	6(11.5)
	No	46(88.5)
Gynecomastia	Yes	1(1.9)
	No	51(98.1)

**Table 4:** Investigation profiles of patients.

Investigation		Frequency (%)
Ascitic gram stain (n=24)	Positive	2(8.3)
	Negative	22(91.7)
Ascitic AFB stain (n=24)	Yes	0(0)
	No	24(100)
Hepatitis B surface antigen (n=52)	Not determined	10(19.2)
	Positive	9(17.3)
	Negative	33(63.5)
Anti-Hepatitis C antibody (n=52)	Not determined	11(21.2)
	Yes	4(7.7)
	No	37(71.2)
Antinuclear antibody (n=52)	Not determined	50(96.2)
	Negative	2(3.8)
HIV test (n=52)	Not determined	39(75)
	Positive	2(3.8)
	Negative	11(21.2)

## Data Availability

The data used to support the findings of this study are available from the corresponding author upon request.

## Abbreviations

AFB: Acid-fast bacilli
ALT: Alanine transaminase
AST: Aspartate transaminase
HBV: Hepatitis B virus
HBsAg: Hepatitis B surface antigen
HCV: Hepatitis C virus
HCT: Hematocrit
HIV: Human-immunodeficiency virus
CKD: Chronic kidney disease
CT: Computerized tomography
CLD: Chronic liver disease
DC: Data collector
ESRD: End-stage renal disease
HF: Heart failure
HSS: Hepatosplenic schistosomiasis
INR: International normalized ratio
NASH: Nonalcoholic steatohepatitis
OPD: Outpatient department
PI: Principal investigator
PLT: Platelet
PT: Prothrombin time
SAAG: Serum ascitic albumin gradient
SBP: Spontaneous bacterial peritonitis
SN: Sensitivity
TB: Tuberculosis
TBP: Tuberculous peritonitis
UoG: University of Gondar
USA: United States of America
WBC: White blood cell.
